# Ba(Zn_1−2*x*_Mn_*x*_Cu_*x*_)_2_As_2_: A Bulk Form Diluted Ferromagnetic Semiconductor with Mn and Cu Codoping at Zn Sites

**DOI:** 10.1038/srep15507

**Published:** 2015-10-23

**Authors:** Huiyuan Man, Shengli Guo, Yu Sui, Yang Guo, Bin Chen, Hangdong Wang, Cui Ding, F.L. Ning

**Affiliations:** 1 Department of Physics, Zhejiang University, Hangzhou 310027, China; 2 Collaborative Innovation Center of Advanced Microstructures, Nanjing 210093, China; 3 Center for Condensed Matter Science and Technology, Department of Physics, Harbin Institute of Technology, Harbin, 150001, China; 4 Department of Physics, Hangzhou Normal University, Hangzhou 310016, China

## Abstract

We report the synthesis and characterization of a bulk form diluted magnetic semiconductor Ba(Zn_1−2*x*_Mn_*x*_Cu_*x*_)_2_As_2_ 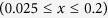 with the crystal structure identical to that of “122” family iron based superconductors and the antiferromagnet BaMn_2_As_2_. No ferromagnetic order occurs with (Zn, Mn) or (Zn, Cu) substitution in the parent compound BaZn_2_As_2_. Only when Zn is substituted by both Mn and Cu simultaneously, can the system undergo a ferromagnetic transition below *T*_*C*_ ~ 70 K, followed by a magnetic glassy transition at *T*_*f*_  ~ 35 K. AC susceptibility measurements for Ba(Zn_0.75_Mn_0.125_Cu_0.125_)_2_As_2_ reveal that *T*_*f*_ strongly depends on the applied frequency with 
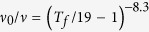
 and a DC magnetic field dependence of 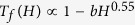, demonstrating that a spin glass transition takes place at *T*_*f*_. As large as −53% negative magnetoresistance has been observed in Ba(Zn_1−2*x*_Mn_*x*_Cu_*x*_)_2_As_2_, enabling its possible application in memory devices.

The successful fabrication of III-V diluted magnetic semiconductors (In, Mn)As and (Ga, Mn)As through low temperature molecular beam epitaxy (LT-MBE) has opened up a new window for the study of magnetic semiconductors^1^,^2^,^3^,^4^,^5^. It is proposed that the Curie temperature would reach room temperature with high enough spin and carrier densities^6^. Nevertheless, the low solid solubility of *Mn*^2+^ for *Ga*^3+^ makes it difficult to enhance the concentration of Mn. As of today, the highest Curie temperature, *T*_*C*_, of (Ga, Mn)As films has been reported as 200 K^7^. On the other hand, *Mn*^2+^ substituting for *Ga*^3+^ introduces not only carriers but also local moments, and some *Mn*^2+^ enter interstitial sites or even As sites, which makes it difficult to separate the charges and spins, and investigate their individual influences on the ferromagnetism. Seeking for new DMS materials that have higher chemical solubility of magnetic atoms and whose carrier density and spin density can be controlled separately may be helpful to improve *T*_*C*_ and understand the mechanism of the ferromagnetic ordering^8^.

Recently, many novel DMSs that are derivatives of Fe-based superconductors have been reported^8^,^9^,^10^,^11^,^12^,^13^,^14^,^15^,^16^,^17^,^18^,^19^. It has been shown from NMR^20^ and *μ*SR measurements that the ferromagnetism in (BaK)(ZnMn)_2_A*s*_2_^9^, Li(Zn, Mn)As^10^, (La, Ba)(Zn, Mn)AsO^12^ and Li(Zn, Mn)P^21^ are homogeneous, i.e., the long range ferromagnetic ordering is arising from the Mn atoms doped at Zn sites, instead of Mn related magnetic impurities. Furthermore, *μ*SR results demonstrated that these bulk form DMSs share the same mechanism for the ferromagnetic ordering as that of (Ga, Mn)As^22^. These bulk form DMSs have the advantages of decoupled spin and carrier doping, and the carrier densities can be controlled and tuned, which overcomes the low carrier densities encountered in II-VI DMS^23^. These systems are isostructural to its variants, i.e., antiferromagnets and superconductors with lattice matching within 5%, which provides the the possibility to make junctions with these materials thorough the As layer^19^. In addition, the bulk form specimens would enable the magnetic techniques to provide complementary information at a microscopic level, such as nuclear magnetic resonance (NMR) and neutron scattering^12^. Among them, the *T*_*C*_ of (Ba, K)(Zn, Mn)_2_As_2_ single crystal has been reported to reach 230 K^24^. (Ba, K)(Zn, Mn)_2_As_2_ was synthesized by doping Mn and K into the parent compound *β*-BaZn_2_As_2_ which is a direct gap (0.2 eV) semiconductor^25^, where the substitution of Mn for Zn and K for Ba introduces spins and hole carriers, respectively.

In this paper, we report the successful fabrication of a new DMS material with a rather new synthesize route, which is different to the previously reported ~10 DMSs^8^,^9^,^10^,^11^,^12^,^13^,^14^,^15^,^16^,^17^,^18^,^19^. Instead of doping at different sites, we co-doped both Mn and Cu into the same Zn sites of BaZn_2_As_2_ to introduce local moments and carriers, respectively. A new series of DMS compounds Ba(Zn_1−2*x*_Mn_*x*_Cu_*x*_)_2_As_2_ (0.025 ≤ *x* ≤ 0.2) have been successfully fabricated. While the system remains semiconducting, 20% Mn and Cu doping results in a ferromagnetic transition below *T*_*C*_ ~ 70 K, followed by a magnetic glassy transition below *T*_*f*_  ~ 35 K. AC susceptibility measurements on an *x* = 0.125 sample indicate that *T*_*f*_ strongly depends on the applied frequencies and magnetic fields, which confirms the spin glass nature at *T*_*f*_. In addition, as large as ~−53% negative magnetoresistance (MR) at a magnetic field *H* = 50 KOe has been achieved in Ba(Zn_0.75_Mn_0.125_Cu_0.125_)_2_As_2_, which is attributed to the suppression of spin fluctuations by magnetic field. Future work is needed to gain deeper understanding of the magnetic behavior of this system and achieve higher *T*_*C*_ values.

## Results and Discussion

### Synthesis and structural characterization

**Figure 1:**
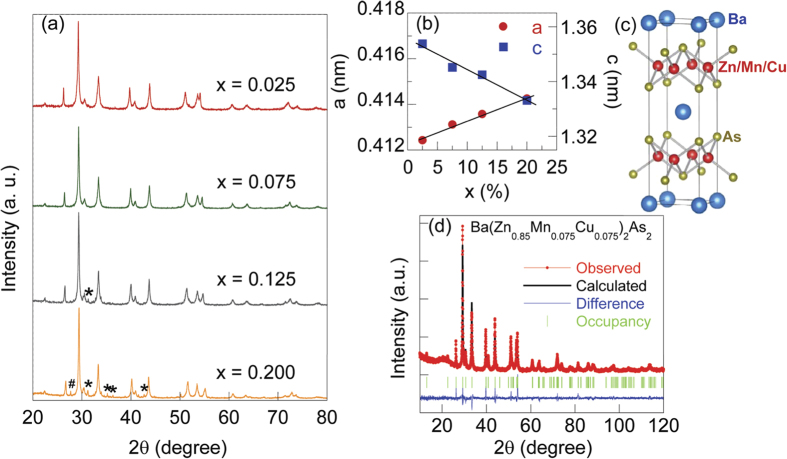
Structural properties of Ba(Zn_1−2*x*_Mn_*x*_Cu_*x*_)_2_As_2_. (**a**) The X-ray diffraction patterns of Ba(Zn_1−2*x*_Mn_*x*_Cu_*x*_)_2_As_2_ (*x* = 0.025, 0.075, 0.125, 0.20). Traces of BaZn_2_As_2_ with space group Pnma (*) and impurity Ba_3_As_14_ (#) are marked; (**b**) The systematic change of the lattice constants *a* (red filled circles) and *c* (blue filled squares) with *x*. (**c**) The layered crystal structure. (**d**) The Rietveld refinement of the powder X-ray diffraction for the *x* = 0.075 sample.

The polycrystalline specimens of Ba(Zn_1−2*x*_Mn_*x*_Cu_*x*_)_2_As_2_ (x = 0.025, 0.075, 0.125, 0.200) were synthesized by the solid state reaction method. Details of the synthesis and facilities used for characterization are described in the Methods section. In Fig. 1, we show the X-ray diffraction patterns for polycrystalline Ba(Zn_1−2*x*_Mn_*x*_Cu_*x*_)_2_As_2_ (0.025 ≤ *x* ≤ 0.200). The Rietveld refinement for Ba(Zn_0.85_Mn_0.075_Cu_0.075_)_2_As_2_ with parameters *R*_*WP*_ = 10.52 %, *R*_*P*_ = 7.58 %, *χ*^2^ = 1.348 shows that the Bragg peaks can be well indexed into the tetragonal structure with space group *I*4/*mmm*. The lattice parameter *a* increases and *c* decreases monotonically with the doping concentration *x*, indicating the successful doping of Mn and Cu into the lattice. We show the crystal structure in Fig. 1(c), which is isostructural to the parent compound of 122-type Fe-based superconductor Ba(Fe_1−*x*_Co_*x*_)_2_As_2_^26^ with *T*_*c*_ = 22 K and antiferromagnet BaMn_2_As_2_ with Néel temperature *T*_*N*_ = 625 K^27^. This feature provides the possibility to make junctions with these systems though As layer. No peaks of impurities are detected for the doping levels of *x* = 0.025 and *x* = 0.075. *α*-BaZn_2_As_2_ with space group of *Pnma* appears for *x* = 0.125 and becomes markable for *x* = 0.20, as marked by * in Fig. 1(a). Small traces of non-magnetic Ba_3_As_14_ impurity are marked as #. Both *α*-BaZn_2_As_2_ and Ba_3_As_14_ are Pauli paramagnetic, which will not affect the magnetic behavior of Ba(Zn_1−2*x*_Mn_*x*_Cu_*x*_)_2_As_2_ discussed in the following.

### Resistivity

**Figure 2:**
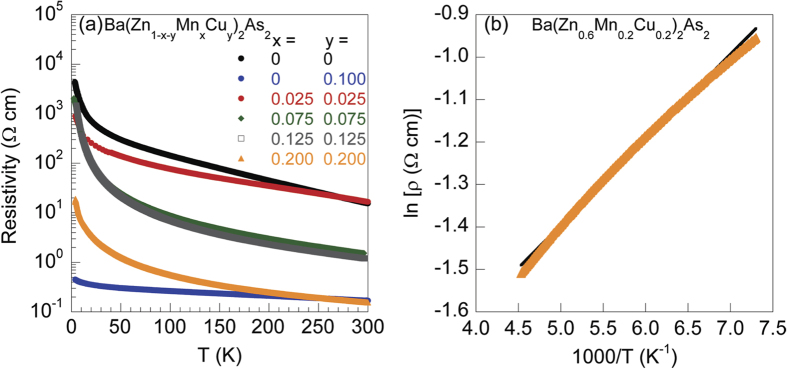
Electrical properties of Ba(Zn_1−2*x*_Mn_*x*_Cu_*x*_)_2_As_2_. (**a**) The temperature dependence of electrical resistivity for BaZn_2_As_2_, Ba(Zn_0.9_Cu_0.1_)_2_As_2_ and Ba(Zn_1−2*x*_Mn_*x*_Cu_*x*_)_2_As_2_ (*x* = 0.025, 0.075, 0.125, 0.20) in logarithmic scale; (**b**) The fit of resistivity for Ba(Zn_0.6_Mn_0.2_Cu_0.2_)_2_As_2_ according to *ρ* ∝ *exp*(*E*_*g*_/2*k*_*B*_*T*).

In Fig. 2, we show the temperature dependent resistivity of the parent compound BaZn_2_As_2_, Ba(Zn_0.9_Cu_0.1_)_2_As_2_ and Ba(Zn_1−2*x*_Mn_*x*_Cu_*x*_)_2_As_2_ (*x* = 0.025, 0.075, 0.125, 0.20). The resistivity of the parent semiconductor BaZn_2_As_2_ displays a typical semiconducting behavior. With 10% Cu doping, the resistivity of Ba(Zn_0.9_Cu_0.1_)_2_As_2_ is heavily suppressed by an order of 4, indicating that carriers are doped. The semiconducting behavior for Mn and Cu codoped case has been conserved for *x* up to 20%, i.e., resistivity continuously increases with temperature decreasing from room temperature down to 4 K. The absolute value of resistivity at 4 K, however, drops from 10^3^ Ω cm for *x* = 0.025 to 10 Ω cm for *x* = 0.20. We roughly fit the resistivity of Ba(Zn_1−2*x*_Mn_*x*_Cu_*x*_)_2_As_2_ (*x* = 0.025, 0.075, 0.125, 0.20) near room temperature in terms of a thermal activation function^13^. Similar approach has also been employed to (La, Sr)(Zn, Mn)AsO^13^. The fitting result for Ba(Zn_0.6_Mn_0.2_Cu_0.2_)_2_As_2_ is shown in Fig. 2(b) as an example. The values of energy gap *E*_*g*_ are between 0.031 and 0.048 eV, which are about an order of magnitude smaller than that of the parent compound BaZn_2_As_2_^25^. We have conducted Hall effect measurement on Ba(Zn_0.75_Mn_0.125_Cu_0.125_)_2_As_2_, but the large resistivity prevents us to accurately determine the carrier density. A preliminary result shows that the carriers are p-type with the concentration in the order of *p* ~ 10^19^ cm^−3^. And the corresponding mobility is estimated to be in the order of 10^−1^ cm^2^V^−1^s^−1^. This value of carrier density is not unusual in bulk form DMSs, which is comparable to that of (Ba_0.9_K_0.1_)(Cd_2−*x*_Mn_*x*_)As_2_^16^ and two orders of magnitude larger than that of Li(Zn, Mn)P^11^, but an order of magnitude smaller than that of (Ba, K)(Zn, Mn)_2_As_2_^9^ and Li(Zn, Mn)As^10^.

### Magnetization and hysteresis

**Figure 3:**
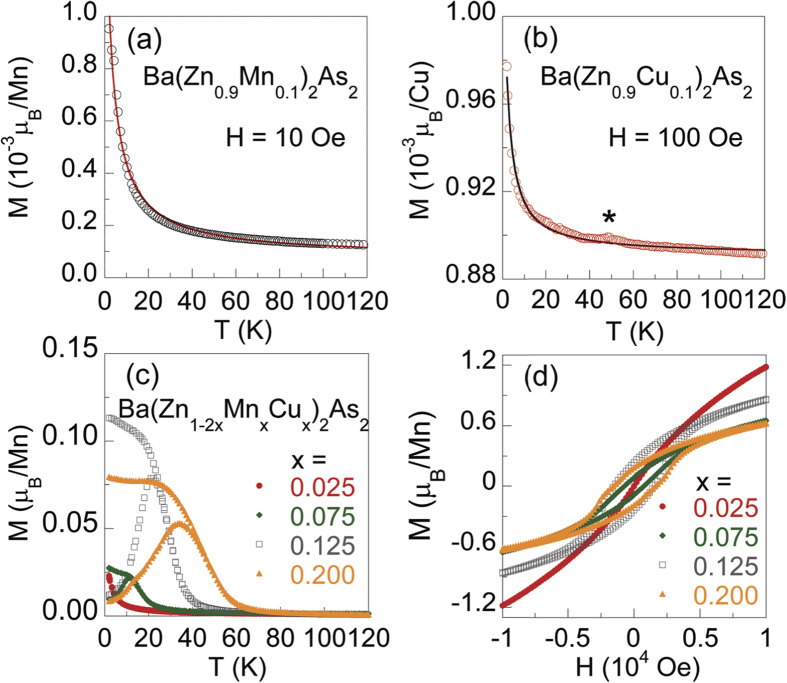
Magnetic properties of Ba(Zn_1−2*x*_Mn_*x*_Cu_*x*_)_2_As_2_. Temperature dependent magnetization *M* for (**a**) Ba(Zn_0.9_Mn_0.1_)_2_As_2_ in an external field of *H* = 10 Oe and (**b**) Ba(Zn_0.9_Cu_0.1_)_2_As_2_ with *H* = 100 Oe. The solid lines represent the Curie-Weiss law *M* = *M*_0_ + *C*/(*T* − *θ*). The star marks the signal from adsorbed oxygen. (**c**) *T*-dependent magnetization *M* for Ba(Zn_1−2*x*_Mn_*x*_Cu_*x*_)_2_As_2_ (*x* = 0.025, 0.075, 0.125, 0.20) in the zero field cooling (ZFC) and field cooling (FC) modes with an external field of *H* = 100 Oe. (**d**) The isothermal magnetization measured at 2 K.

In Fig. 3(a,b), we show the temperature dependence of magnetization for Ba(Zn_0.9_Mn_0.1_)_2_As_2_ and Ba(Zn_0.9_Cu_0.1_)_2_As_2_, respectively. No anomaly or transition is observed in the measured temperature range, and the moment at 2 K is only ~0.001 *μ*_*B*_/(Mn or Cu atom) for both Ba(Zn_0.9_Mn_0.1_)_2_As_2_ and Ba(Zn_0.9_Cu_0.1_)_2_As_2_. We fit the magnetization data to a Curie-Weiss law *M* = *M*_0_ + *C*/(*T* − *θ*) and obtained *C* = 0.00456 *μ*_*B*_K/Mn, *θ* = −2.74 K for Ba(Zn_0.9_Mn_0.1_)_2_As_2_, and *C* = 0.00028 *μ*_*B*_K/Cu, *θ* = −1.45 K for Ba(Zn_0.9_Cu_0.1_)_2_As_2_, indicating the paramagnetic ground state. These results indicate that doping either Mn or Cu alone into BaZn_2_As_2_ can not form any type of magnetic ordering. This feature has also been observed in LaZnAsO, where doping Mn or Fe only does not result in ferromagnetic ordering^8^,^12^. The magnetic character of Cu in 122-type arsenides has been investigated by density functional calculations^28^ and intensive transport properties measurements^29^. Cu 3*d* bands are ~3 eV below Fermi energy (*E*_*F*_), and contribute little to the density of states at *E*_*F*_^28^. The 3*d* shell of Cu is completely filled with 3*d*^10^ electronic configurations^28^,^29^. Therefore, the valence of Cu in 122-type arsenides is +1 with nonmagnetic state S = 0^28^,^29^. The paramagnetic state of Ba(Zn_0.9_Cu_0.1_)_2_As_2_ are consistent with the previous reports^28^,^29^.

In Fig. 3(c), we show the magnetization of Ba(Zn_1−2*x*_Mn_*x*_Cu_*x*_)_2_As_2_ (*x* = 0.025, 0.075, 0.125, 0.20) with the same amount of Mn and Cu atoms doped into Zn sites of BaZn_2_As_2_. No magnetic transition has been observed for *x* = 0.025. A fit to Curie-Weiss law *M* = *M*_0_ + *C*/(*T* − *θ*) shows that *θ* = −0.6 K, indicating the paramagnetic ground state. For the doping level of *x* = 0.075, a strong increase of magnetization at Curie temperature *T*_*C*_ = 33 K and a bifurcation of zero field cooling (ZFC) and field cooling (FC) curves at spin freezing temperature *T*_*f*_ = 12 K are observed. *T*_*C*_ and *T*_*f*_ are enhanced with increasing doping levels. With 20% doping, *T*_*C*_ increases to 70 K and *T*_*f*_ increases to 35 K. In Fig. 3(d), we present the results of isothermal magnetization measurements. For *x* ≥ 0.075, clear hysteresis loops have been observed at 2 K. The coercive field becomes lager for higher *x*, and reaches 1600 Oe for *x* = 0.20. We should note that this value is much smaller than ~10^4^ Oe of (Ba, K)(Zn, Mn)_2_As_2_^9^. The contrasting ground states shown in Fig. 3(a,b,c) unequivocally demonstrate that only when Zn is substituted by both Mn and Cu simultaneously, can the ferromagnetic ordering develop, which also indicates that the ferromagnetic signals result from the doping of Mn and Cu rather than impurities.

We fit the *T*-dependent magnetization above *T*_*C*_ to the Curie-Weiss formula *χ* = *χ*_0_ + *C*/(*T* − *θ*) in order to obtain the Weiss temperature (*θ*) and effective paramagnetic moment of Mn (*μ*_*eff*_). The best fittings show that the effective moment *μ*_*eff*_ is 4.8 ~ 5.7 *μ*_*B*_/Mn for 0.025 ≤ *x* ≤ 0.20, indicating the high spin state of Mn with the valence of +2 in the system of Ba(Zn_1−2*x*_Mn_*x*_Cu_*x*_)_2_As_2_. We tabulate the Curie temperature *T*_*C*_, the spin freezing temperature *T*_*f*_ (the temperature where ZFC and FC curves split), the base temperature moment *μ*_*BT*_ (the values at 2 K measured from FC curves with *H* = 100 Oe), the coercive field *H*_*c*_ and the energy gap *E*_*g*_ (fitted from the resistivity data) in Table 1. *T*_*C*_, *T*_*f*_, *θ* and *H*_*c*_ show a trend of increasing with higher doping level *x*, indicating the strengthening of ferromagnetic exchange interaction with higher concentration of Mn and Cu. Meanwhile, the systematic changes of these magnetic parameters suggest that the magnetic signals in this system are not caused by impurities. On the other hand, we notice that *μ*_*BT*_ first increases from 0.027 *μ*_*B*_/Mn for *x* = 0.075 to 0.110 *μ*_*B*_/Mn for *x* = 0.125, but decreases to 0.079 *μ*_*B*_/Mn for *x* = 0.20. This may be due to the competition of ferromagnetic and antiferromagnetic exchange interactions between Mn atoms.

To further investigate the valence of Cu and Mn, we conducted the X-ray photoelectron spectroscopy (XPS) measurements for Ba(ZnMn_0.2_Cu_0.2_)_2_As_2_. Ba and Zn have been observed from the peaks of binding energy. But unfortunately, after very careful comparison, we haven’t detected effective peaks of Cu or Mn from the binding energy. No conclusion about the valence of Cu or Mn has been achieved from the XPS measurements. We can’t obtain evidence from XPS that whether Cu contribute magnetic moments or not. Magnetic resonance techniques may be used in the future to further investigate the magnetic mechanism of this system.

### Magnetoresistance

**Figure 4:**
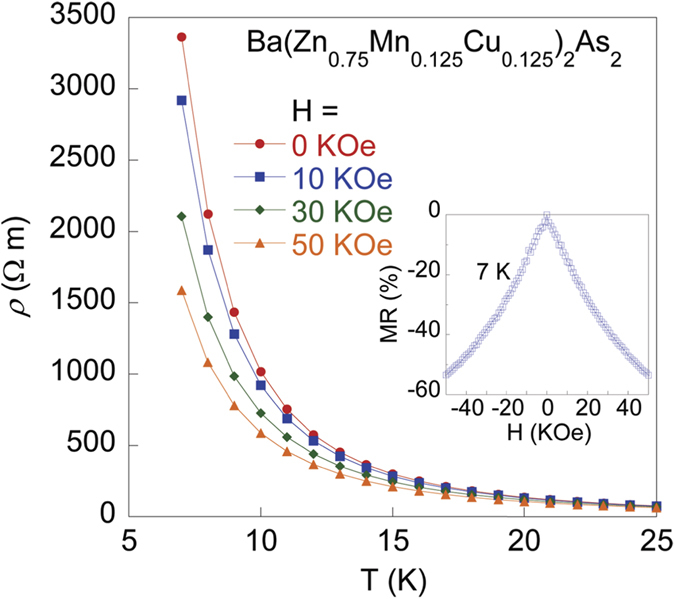
*T*-dependent magnetoresistivity for Ba(Zn_0.75_Mn_0.125_Cu_0.125_)_2_As_2_ under *H* = 0, 10, 30, 50 KOe. The inset shows the field dependence of magnetoresistance at 7 K from −50 KOe to 50 KOe.

We measured the magnetoresistance for Ba(Zn_0.75_Mn_0.125_Cu_0.125_)_2_As_2_ under the applied fields of 0, 10, 30, 50 KOe, and show the results in Fig. 4. The resistivity with different fields deviates from each other at ~22 K, and the values of *ρ* at 7 K monotonically drop from 3363 Ω m at 0 Oe to 1587 Ω m at 50 KOe. The magnetoresistance (defined as [*ρ*(*H*) − *ρ*(0)]/*ρ*(0) at 7 K) reaches −53 % at 50 KOe. The large negative magnetoresistance has also been observed in other bulk form DMSs, such as (Ba_0.9_K_0.1_)(Cd_2−*x*_Mn_*x*_)_2_As_2_^16^ and (Sr_0.9_K_0.1_)(Zn_1.8_Mn_0.2_)As_2_^17^. We tentatively attribute the negative magnetoresistance to the suppression of spin fluctuations by applied field.

### AC susceptibility

**Figure 5:**
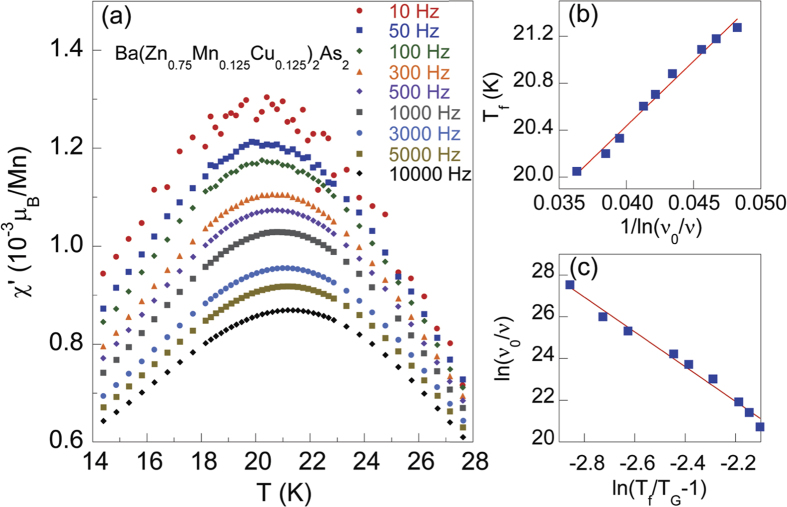
AC susceptibility at various frequencies for Ba(Zn_0.75_Mn_0.125_Cu_0.125_)_2_As_2_. (**a**) The real component 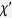 of AC magnetic susceptibility as a function of temperature *T* measured from 10 Hz to 10000 Hz. (**b**) Spin freezing temperature *T*_*f*_ as a function of 1/*ln*(*ν*_0_/*ν*) fitted with the Vogel-Fulcher law. (**c**) A linear fit of *ln*(*ν*_0_/*ν*) versus *ln*(*T*_*f*_/*T*_0_ − 1) with Eq. (2).

We measured the AC susceptibility, 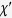, for the *x* = 0.125 sample at various frequencies *ν* under zero external field, and show the results in Fig. 5. We found that the maxima of the real part, 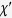, drop obviously, and *T*_*f*_ shifts slightly to higher temperature with the increasing AC frequencies. This feature is typically taken as signs for spin glass systems^30^,^31^,^32^,^33^,^34^,^35^,^36^,^37^. This kind of behavior has also been observed in CaNi_1−*x*_Mn_*x*_Ge^30^, CeCu_4_Mn^31^, La(Fe_1−*x*_Mn_*x*_)_1.4_Si_1.6_^32^ and II-VI family DMS^23^. The Vogel-Fulcher law^38^,^39^,^40^,^41^ is usually used to describe the dependence between *T*_*f*_ and *ν*,


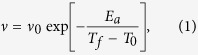


where *E*_*a*_ is the activation energy, *T*_0_ is the Vogel-Fulcher temperature, and *ν*_0_ is the fitted frequency. We tried different values of *ν*_0_ from 10^10^ Hz to 10^13^ Hz, which showed that the best linear fitting can be obtained when 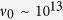 Hz, in good agreement with expectation for a spin-glass 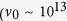 Hz) rather than a cluster-glass 
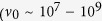
 Hz)^42^. So *ν*_0_ is considered as a constant value of 10^13^ Hz for this system in the following discussion. In Fig. 5(b), *T*_*f*_ is plotted as a function of 1/*ln*(*ν*_0_/*ν*). The well fitted linear relation enables us to estimate the value of *T*_0_ and *E*_*a*_. *T*_0_ is ~16 K and *E*_*a*_ is ~110.48 K, corresponding to 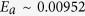 eV because of *E* = *k*_*B*_*T* with 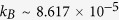 eV/K. So *E*_*g*_ = 2*E*_*a*_ = 0.02 eV, which is in the same order of magnitude as the value estimated from the fit of resistivity. The ratio 
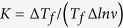
 is usually used to distinguish the frequency sensitivity of *T*_*f*_ in a spin glass^40^,^43^. *K* is the order of 0.01 for spin glass systems, while *K*  > 0.1 for superparamagnets^36^. For the *x* = 0.125 sample, *K* is estimated to be ~0.008 ± 0.002, in good agreement with the typical values reported for spin glasses^30^,^31^,^37^,^40^.

The dynamical slowing down of spin fluctuations can also be expressed by the standard power dependence,


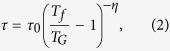


where *τ* = 1/*ν* is the relaxation time, *τ*_0_ = 1/*ν*_0_ is set as 10^−13^ s, *T*_*G*_ is the spin freezing temperature, *η* is the dynamic exponent. When *T*_*f*_ approaches *T*_*G*_ which is the zero frequency limit, the order of *τ* gets much larger than *τ*_0_, indicating that spin fluctuations significantly slow down. A linear fit of *ln*(*ν*_0_/*ν*) versus *ln*(*T*_*f*_/*T*_*G*_ − 1) according to Eq. (2) is shown in Fig. 5(c), yielding *T*_*G*_ ~ 18.96 K and 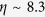. The value of *η* falls into the range of 4–12 for spin glasses^31^,^32^,^33^,^36^,^40^,^44^,^45^, which is not cluster-glass like character^46^. 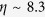 is close to 7.9, the calculated value for the three-dimensional Ising spin-glass^47^,^48^.

**Figure 6:**
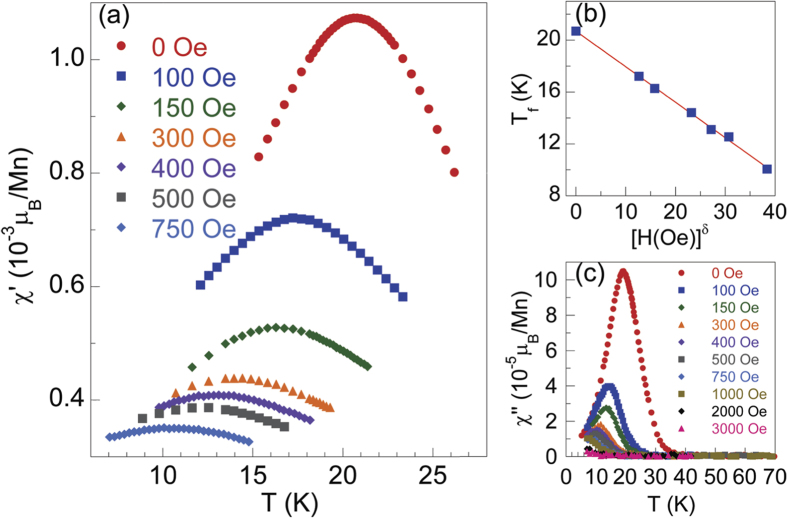
AC susceptibility with various DC fields for Ba(Zn_0.75_Mn_0.125_Cu_0.125_)_2_As_2_. (**a**) The temperature dependence of the AC susceptibility 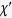 measured at a frequency of 500 Hz under different applied DC fields. (**b**) *T*_*f*_ as a function of *H*^*δ*^ with *δ* = 0.55. The straight line is a guide for eyes. (**c**) *T*-dependent imaginary part 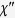 at 500 Hz with various DC fields from 0 to 3000 Oe.

In Fig. 6(a), we show the measurements of *T*-dependent AC susceptibility at a fixed frequency of 500 Hz with various DC fields for Ba(Zn_0.75_Mn_0.125_Cu_0.125_)_2_As_2_. The AC susceptibility is strongly affected by the external DC fields, i.e., the cusps smear out, the peak value of 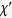 decreases remarkably, and *T*_*f*_ shifts to lower temperature with increasing DC fields. These are all characteristic features of spin glasses^30^,^32^,^49^. The DC field dependence of the spin freezing temperature *T*_*f*_ can be described by the equation,


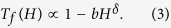


A best fit of *T*_*f*_ versus *H* to Eq. (3) yields 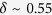. We show the plot of *T*_*f*_ versus *H*^0.55^ in Fig. 6(b). *δ* is ~2/3 for Ising spin glass systems, and *δ* = 2 for Heisenberg systems^50^,^51^. In the current case, *δ* is close to 2/3, indicating that the glassy state for Ba(Zn_1−2*x*_Mn_*x*_Cu_*x*_)_2_As_2_ may be explained by mean-field theory with Ising model. In Fig. 6(c), we show the imaginary component of AC susceptibility at 500 Hz with DC fields up to 3000 Oe. Similar to the case of 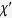, *T*_*f*_ decreases noticeably with increasing fields. The *T*-dependent imaginary part 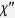 of the AC susceptibility under 2000 Oe and 3000 Oe becomes almost undependent of *T*.

## Conclusion

A bulk form diluted magnetic semiconductor Ba(Zn_1−2*x*_Mn_*x*_Cu_*x*_)_2_As_2_ (0.025 ≤ *x* ≤ 0.2) with maximum *T*_*C*_ ~ 70 K has been successfully synthesized. It is the first time that ferromagnetic ordering is observed when Mn and Cu are codoped into the Zn sites, where Mn substitution for Zn introduces spin and Cu substitution for Zn introduces carriers, respectively. The new system displays large negative magnetoresistance while conserving the semiconducting behavior with the doping level up to 20%. The AC susceptibility measurements show that the spin freezing temperature *T*_*f*_ is dependent on frequency and external field, confirming the glassy nature below 35 K. Finally, the new DMS system has a tetragonal crystal structure identical to that of “122” family of Fe-based superconductors and the antiferromagnetic system BaMn_2_As_2_, which makes it possible to make various junctions of these systems through As layer. More theoretical and experimental work are expected to further understand the properties and physics of this system.

## Methods

The polycrystalline specimens of Ba(Zn_1−2*x*_Mn_*x*_Cu_*x*_)_2_As_2_ (*x* = 0.025, 0.075, 0.125, 0.200) were synthesized by the solid state reaction method. High purity elements of Zn (99.9%), Mn (99.99%), Cu (99.9%) and As (99%) were mixed, ground and pressed into pellets. The pellets were sealed in evacuated silica tubes and sintered at 800 °C for 10 hours to make the precursors (Zn_1−2*x*_Mn_*x*_Cu_*x*_)As. The mixture of Ba (99.2%) and (Zn_1−2*x*_Mn_*x*_Cu_*x*_)As were then slowly heated to 900 °C and held for 10 hours, then 1150 °C for 15 hours before cooling down to room temperature with the furnace turned off. The handling of materials were performed in a high-purity argon filled glove box (the percentage of *O*_2_ and *H*_2_*O* ≤ 0.1 ppm) to protect it from exposure to air. Powder x-ray diffraction was performed at room temperature using a PANalytical x-ray diffractometer (Model EMPYREAN) with monochromatic *CuK*_*α*1_ radiation. The electrical resistance was measured on sintered pellets with the typical four-probe method. The DC magnetization measurements were conducted on a Quantum Design Magnetic Property Measurement System (MPMS-5). The AC susceptibility and magnetoresistance were measured on a Quantum Design Physical Property Measurement System (PPMS-9).

## Additional Information

**How to cite this article**: Man, H. Y. *et al.* Ba(Zn_1-2x_Mn_x_Cu_x_)_2_As_2_: A Bulk Form Diluted Ferromagnetic Semiconductor with Mn and Cu Codoping at Zn Sites. *Sci. Rep.* **5**, 15507; doi: 10.1038/srep15507 (2015).

## Figures and Tables

**Table 1:** Curie temperature (*T*_*C*_), spin freezing temperature (*T*_*f*_), Weiss temperature (*θ*), base temperature moment (*μ*_*BT*_, the values at 2 K from FC curves with *H* = 100 Oe), the effective moment (*μ*_*eff*_), coercive field (*H*_*c*_), and energy gap (*E*_*g*_, fitted from resistivity) for Ba(Zn_1−2*x*_Mn_*x*_Cu_*x*_)_2_As_2_ (0.025 ≤ *x* ≤ 0.20).

*x*	*T*_*C*_(K)	*T*_*f*_(K)	*θ*(K)	*μ*_*BT*_(*μ*_*B*_/Mn)	*μ*_*eff*_(*μ*_*B*_/Mn)	*H*_*c*_(Oe)	*E*_*g*_(eV)
0.025	—	—	−0.6	0.023	5.1	0	0.031
0.075	33	12	12.9	0.027	4.8	730	0.039
0.125	44	22	31.7	0.110	5.7	1528	0.048
0.20	70	35	50.1	0.079	5.5	1600	0.035
